# Sexual knowledge and contraceptive use in adolescents with type 1 diabetes in comparison with their healthy peers

**DOI:** 10.1007/s12020-025-04345-1

**Published:** 2025-08-31

**Authors:** K. Karavanaki, K. Kakleas, B. Kandyla, A. Soldatou, G. Paltoglou, S. E. Karanasios, C. Tzavara, A. Tsitsika, L. Kossiva

**Affiliations:** 1 Diabetes Unit, Second University Department of Pediatrics, “P. & Α. Kyriakou” Children’s Hospital, National and Kapodistrian University of Athens School of Medicine, Athens, Greece; 2 First University Department of Pediatrics, “Aghia Sophia” Children’s Hospital, National and Kapodistrian University of Athens School of Medicine, Athens, Greece; 3 Adolescent Health Unit (Α.H.U.) Second University Department of Pediatrics, “P. & Α. Kyriakou” Children’s Hospital, National and Kapodistrian University of Athens School of Medicine, Athens, Greece

**Keywords:** Sexual education, Contraception, STDs, Adolescents, Type 1 diabetes

## Abstract

**Purpose:**

Adolescents with type 1 diabetes mellitus (T1D) may differ from their healthy peers in respect to sexually transmitted diseases (STDs) knowledge and contraceptive use. We aimed to explore sexual knowledge and contraceptive use and associated factors in T1D adolescents compared to healthy peers.

**Methods:**

Fifty- eight T1D adolescents (mean ± SD age 16.3 ± 2.0 years, disease duration 6.7 ± 3.5 years) were compared to 116 healthy controls (matching 1:2 for school, age and gender). Anonymous questionnaires were used to evaluate sexual knowledge and contraceptive methods.

**Results:**

The commonest contraceptive method at the last sexual contact was the condom. The use of birth pill was reported by 11.8% patients and by 8.3% controls, withdrawal was reported by 33.3% of T1D and 24% of controls, no protection by 23.5% patients and by 10.2% controls, while double protection (≥2 methods) was reported by 35% patients and 27.7% controls. No study participant used long-acting reversible contraception (LARC). The high protection (dual protection) group was characterized by younger age and older age at sexual debut (16.4 vs 15.8 years, *p* = 0.010). The low protection group (no contraception/withdrawal) was characterized by older patients’ age (*p* = 0.023) and younger paternal age (*p* = 0.046). Among controls, the use of dual protection was more common in the group whose parents were married versus those with divorced parents (34.3 vs. 10%, *p* = 0.042).

**Conclusions:**

Among the study population, the condom was the commonest contraceptive method for both groups, while 23.5% of patients and 10.2% controls used no protection. The degree of contraception use among patients was associated with patients’ age and parental age and with the family structure in controls. The above underline the necessity for health care professionals to provide sexual education and contraception counseling to young adolescents with T1D and their healthy peers.

## Introduction

Type 1 diabetes (T1D) is a complex chronic illness that needs a holistic approach based on insulin use, dietary modifications, and exercise. It influences patients’ quality of life and has particular implications for adolescents and their families. Deterioration of metabolic control during adolescence is a common issue [1].

It has been reported that 50% of all teenagers aged 15–19 years are sexually active. Falsetti et al. reported no difference in the prevalence of unplanned pregnancy between 87 female adolescents with T1D and 45 non-diabetic adolescents aged 16–22 years (4.6 vs 4.2%, respectively). In contrast, 3.5% of adolescents with T1D suffered from a sexually transmitted disease (*Chlamydia* or *Trichomonas*) [2]. Thus, adolescent females, with or without T1D, exhibited less than optimally protected sexual activity, which increased the risk of unplanned pregnancies and sexually transmitted infectious diseases.

Regarding the use of contraceptive methods among T1D adolescents, a study of 89 female adolescents with T1D revealed that only half used condoms or other measures of birth control [3]. At the same time, 61% of the general population do so [4]. The condom is the preferred contraceptive method for adolescents with T1D [3, 5, 6]. Among adolescents with T1D, oral contraceptives seem to be the second method of choice [2, 3, 6]. According to WHO, adolescents with diabetes less than 20 years old could use any method of contraception [7]. Moreover, according to the recommendations from the American College of Obstetricians and Gynecologists [8] and the American Academy of Pediatrics, long-acting reversible contraceptive methods (LARCs), including intrauterine devices and subdermal progestin implants, should be considered a first-line choice for contraception in adolescents and young women with T1D and T2D.

To our knowledge, there is limited experience among Gynaecologists about contraception in young women with T1D, and there are also few relative previous studies in the literature. Moreover, poor glycemic control before conception and early in pregnancy increases the risk for maternal and fetal complications [2]. For the above reasons, the use of birth control measures and sexual education is of utmost importance in T1D adolescents.

The purpose of this study was to assess the sexual knowledge and the use of different contraceptive methods in T1D adolescents and associated factors in comparison with their healthy peers.

## Materials and methods

### Materials

The study group included 58 T1D adolescents (29 boys) (mean age 16.3 ± 2years,) who were followed by the Diabetes Unit of the Second Department of Pediatrics “P.&Α.Kyriakou” Children’s Hospital, National and Kapodistrian University of Athens for 4 years (2015–2019) with a disease duration of 6.7 ± 3.5 years (range:2–15.5 years) and a mean annual HbA1c of 8.01 ± 1.3% (range: 6.1–11.4%) and 116 healthy controls (58 boys) matched for educational level, gender, age and socio-economic level with a mean ± SD age of 15.8 ± 1.4 years. A detailed population description can be found in a previous article, [9].

The study was approved by the Ethics Committee of the “P. & A. Kyriakou” Children’s Hospital in Athens, Greece and was in accordance with the Helsinki declaration. Informed consent was obtained from the parents or legal guardians of all the participants.

### Methods

The collected data included the demographic characteristics of the participants, place of residence, socioeconomic status and parents’ education and occupation. For every T1D patient included, a detailed personal medical questionnaire was completed, focusing on diabetes duration and type of insulin therapy. In both the patient group and the control group, somatometric parameters (weight, height, BMI), blood pressure, and heart rate were recorded. The HbA1c level (mean value) over the last year of the patient group was also recorded. Anonymous self-completed questionnaires were used to evaluate: a) sexual activity and behavior, b) the use of a contraceptive method, and c) other psychosocial factors.

A questionnaire, that has been used previously in studies of sexual activity and contraception methods among healthy Greek adolescents [10, 11], contained 72 questions assessing:

- *Socio-demographic variables*. Participants’ age, gender, nationality and parental educational and occupational status, measured by the highest qualification earned by either parent. Additionally, parental marital status (married, separated/divorced, widowed) were recorded for all study participants.
- *Sexual activity*. Sexual experience was defined as any sexual contact, excluding vaginal sexual intercourse (e.g. hugging and mouth kissing, oral sex etc). Sexual intercourse was defined as vaginal sexual intercourse on at least one occasion.
- *Contraceptive use*. Study participants were also requested to report the contraceptive methods used during sexual encounters. The contraceptive methods were as follows: i) no protection applied during sexual encounter; ii) withdrawal of penis prior to ejaculation; iii) avoiding sexual intercourse during possible ovulation days («rhythm method»); iv) condom use by male partner; v) oral contraceptive use by female partner; vi) intrauterine devices, vii) subdermal progestin implants, viii) next-day’s pill used by female partner.
- *Sources of sexual information*. The sources providing sexual information to the adolescents included the following categories: i) school environment, ii) Pediatrician, Gynecologist or other physician, iii) siblings, iv) friends, v) television shows, vi) books related to sexual health, vii) magazines, and viii) internet sites related to sex, ix) church.
- Additional *Psychosocial* factors were evaluated as follows: i) family status, ii) traumatic or other major life event, iii) peer influence upon sexual initiation and iv) being forced to have coital experience.

### Statistical analysis

The Kolmogorov-Smirnov test was used to estimate the normal distribution of quantitative variables. Quantitative variables are expressed as mean values (SD) or median values (interquartile range). Qualitative variables are expressed as absolute and relative frequencies. Chi-square tests and Fisher’s exact tests were used to compare proportions. Student’s t-tests were computed for comparing mean values when the distribution was normal and the Mann-Whitney test for the comparison of median values was used when the distribution was not normal. Kruskall-Wallis one way ANOVA was used for comparison of quantitative variables among 3 subgroups. Chi-square tests were used for comparison of categorical variables between different subgroups. Logistic regression was used for finding factors associated with low protection and condom use. Odds ratios (OR) with 95% confidence intervals (95% CI) were computed from the logistic regression analyses). All *p* values reported are two-tailed. Statistical significance was set at 0.05 and analyses were conducted using SPSS (IBM SPSS Statistics for Windows, Version 19.0. Armonk, NY: IBM Corp.

## Results

Fifty-eight T1D adolescents with mean ± SD age of 16.3 ± 2 (range: 14–22) years were compared to 116 healthy controls with a mean age of 15.8 ± 1.4 (range: 14–21) years. The two study groups were similar in terms of their parental educational level and marital status, the number of siblings, and nationality, while paternal and maternal age were higher in the T1D group (father: 51.3 ± 9.8 years vs 48.4 ± 6.7years, *p* = 0.026), (mother:46.3 ± 9.4 vs 43.8 ± 4.9 years, *p* = 0.029).

### Sexual knowledge and education

Both study groups had the same knowledge of the reasons for requiring contraception during intercourse. Almost half of T1D adolescents (52.7%) and 41.7% of controls (*p* = 0.088) reported using prophylaxis (contraception) to avoid sexually transmitted diseases.

Regarding knowledge of the participants on sexually transmitted diseases (STD), T1D adolescents reported that HIV is a STD in a significantly lower percentage than controls (76.4 vs. 89.6%, *p* = 0.027), with no significant difference regarding the identification of other diseases as STDs. Only half of the patients and controls knew that syphilis is a sexually transmitted disease (T1D vs controls: 47.8 vs 56.4%, *p* = 0.488). In contrast, the corresponding percentages for the other diseases (e.g. Hepatitis B, C, HPV virus) were lower in both groups (T1D vs controls: HepB: 23.5 vs 36.4%, *p* = 0.180, HepC: 17.4 vs 25.5%, *p* = 0.422, HPV: 15.7 vs 16.4%, *p* = 0.947).

### Contraceptive methods

The frequency of sexual activity was 38/58 (65.5%) among T1D adolescents and 81/116 (69.8%) among controls. A comparison of different contraceptive methods used by adolescents (Table 1) showed no significant differences between the two study groups. It is noteworthy that 34/38 (89.5%) of the sexually active T1D adolescents and 76/81 (93.8%) of the sexually active controls used any contraceptive method, while 4/38 (10.5%) of T1D adolescents and 5/81 (6.2%) of the controls used no protection at all. Moreover, 5/38 (13.1%) sexually active T1D adolescents and 14/81 (17.3%) controls used a combination of two methods (high protection group). The most common method of contraception for the first and most recent sexual intercourse was the condom for both groups. Thus, 85.0% of T1D adolescents and 72.5% of controls used it during their first sexual intercourse, and 71.4% of T1D and 73.1% of controls used it during the most recent intercourse. However, only 47.8% of T1D adolescents and 51.9% of controls reported using condoms during every sexual encounter. It is noteworthy that none of the study participants used intrauterine devices of subdermal progestin implants (long-acting reversible contraception, LARC).

**Table 1:** Contraception methods used by adolescents of the two study groups

	Group		*P*
	Controls	TID	
	*N* = 90 (%)	*N* = 38 (%)	
1a. Contraception method during 1st sexual intercourse used by study participants or partners			
No protection	7 (13.7)	1 (6.3)	0.699**
Withdrawal	16 (30.8)	6 (35.3)	0.783*
Condom	37 (72.5)	17 (85.0)	0.736**
Birth pill	5 (10.0)	3 (18.8)	0.573**
«Rhythm» method	5 (10.0)	2 (12.5)	0.155**
Emergency contraception	4 (8.2)	0 (0.0)	0.555**
Subdermal progestin implants	0 (0)	0 (0)	–
Intrauterine devices	0 (0)	0 (0)	–
Condom plus Withdrawal	8 (18.6)	4 (26.7)	0.487**
Condom plus Birth pill	4 (9.3)	2 (13.3)	0.643**
Condom plus «Rhythm» method	4 (9.3)	2 (18.24)	0.590**
Double protection (≥2 methods)	15 (31.3)	5 (25)	0.606*
Low protection (withdrawal/no protection)	17 (37.0)	6 (37.5)	0.969*
1b. Contraception method during the most recent sexual intercourse used by study participants or partners			
	*N* = 81(%)	*N* = 38(%)	
No protection	5 (10.2)	4 (23.5)	0.430**
Withdrawal	12 (24.0)	6 (33.3)	0.728**
Condom	38 (73.1)	15 (71.4)	1.000*
Birth pill	4 (8.3)	2 (11.8)	0.561**
«Rhythm» method	6 (12.5)	3 (18.8)	0.749**
Emergency contraception	2 (4.3)	3 (18.8)	0.195**
Condom plus Withdrawal	5 (12.2)	2 (14.3)	1.000**
Subdermal progestin implants	0 (0)	0 (0)	–
Intrauterine devices	0 (0)	0 (0)	–
Condom plus Birth pill	3 (7.3)	2 (15.4)	0.584**
Condom plus «Rhythm» method	6 (18.2)	1 (11.1)	1.000**
Double protection (≥2 methods)	13 (27.7)	7 (35.0)	0.548*
Low protection (withdrawal/no protection)	13 (30.2)	7 (43.8)	0.329*
1c. Reason of choice of contraception method at 1st sexual intercourse			
Easy access	11 (20.4)	4 (17.4)	0.530**
Low cost	7 (13.0)	5 (21.7)	0.383**
Avoiding pregnancy	28 (51.9)	12 (52.2)	0.485*
Protection from STDs	26 (48.1)	14 (60.9)	0.422*
No specific reason	8 (14.8)	3 (13.0)	0.471**

*Pearson’s chi-square test

**Fisher’s exact test

Most teenagers used the condom during the 1st sexual intercourse to a. avoid STDs (T1D group 60.9%, controls 48.1%) and b. avoid pregnancy (T1D group 52.2%, controls 51.9%) (Table 1c). Low protection (withdrawal/no protection) at first intercourse was reported by 17 controls (37%) and 6 T1D patients (37.5%), *p* = 0.969.

### Analysis of contraceptive methods by gender

In male adolescents, no significant differences were found in the contraceptive methods used by controls or T1D males, respectively (*p* > 0.05) (Table 2). No significance could be computed in females due to the small sample size.

**Table 2:** Contraceptive methods by group, separately for males and females

Contraceptive methods during the most recent intercourse		Males			Females		
		T1D		P+	T1D		P+
		No	Yes		No	Yes	
		N (%)	N (%)		N (%)	N (%)	
None	No	24 (85.7)	9 (69.2)	0.103	13 (92.9)	2 (100)	–
	Yes	4 (14.3)	4 (30.8)		1 (7.1)	0 (0)	
Withdrawal	No	21 (75)	9 (69.2)	0.733	10 (66.7)	1 (33.3)	–
	Yes	7 (25)	4 (30.8)		5 (33.3)	2 (66.7)	
Condom	No	5 (16.1)	3 (21.4)	0.206	2 (14.3)	0 (0)	–
	Yes	26 (83.9)	11 (78.6)		12 (85.7)	4 (100)	
Birth pill	No	24 (85.7)	9 (81.8)	0.697	13 (100)	2 (100)	–
	Yes	4 (14.3)	2 (18.2)		0 (0)	0 (0)	
«Rhythm» method	No	24 (88.9)	9 (81.8)	0.733	10 (76.9)	1 (50)	–
	Yes	3 (11.1)	2 (18.2)		3 (23.1)	1 (50)	
Emergency contraception	No	24 (92.3)	9 (75)	0.448	13 (100)	2 (100)	–
	Yes	2 (7.7)	3 (25)		0 (0)	0 (0)	
Intrauterine devices	No	28 (100)	0 (0)	–	15 (0)	0 (0)	–
Progestin implants	No	28 (100)	0 (0)	–	15 (0)	0 (0)	

The data of the table referred only in those who had a complete sexual intercourse

+*p*-value from conditional logistic regression

### Degree of contraception use by demographic variables

The patients and control groups were divided into three categories according to the use of one contraceptive method (ordinary group), two or more contraceptive methods (high protection group), or no contraception/rhythm method (low protection group). Thus, there were 7 (35%) T1D adolescents and 13 (27.7%) controls in the double protection group. Moreover, 7 (43.8%) T1D adolescents and 13 (30.2%) controls were in the low protection group. The effect of different demographic variables on the degree of contraception use in patients with T1D is shown in Table 3 and controls in Table 4.

**Table 3:** a Patients with T1D-level of contraception by demographic parameters. b level of contraception according to parental educational status

	HIGH protection (a) *n* = 6 (≥2 methods)	ORDIΝARY protection (b) *n* = 13 (1 method)	LOW PROTECTION (c) *n* = 2 (no contraception or withdrawal)	*p*
a				
Patient’s age (years)	**16.5** ± **1.04**	18.19 ± 2.76	**17.0** ± **2.82**	0.343*
	a vs b → *p* = 0.077 a vs c → ***p*** = 0.023**			
Age at T1D diagnosis (years)	9.12 ± 3.64	9.8 ± 1.75	9.39 ± 0.0	0.845*
	a vs b → *p* = 0.060**			
Age at 1st sexual contact (years)	**16.40** ± **0.548**	**15.77** ± **2.22**	15.5 ± 0.707	0.779*
	a vs b → ***p*** = 0.010** b vs c → *p* = 0.093**			
Father’s age (years)	48.67 ± 7.55	**51.92** ± **5.89**	**41.0** ± **14.14 years**	0.140*
	b vs c → ***p*** = 0.046**			
Mother’s age (years)	46 ± 5.0	47.58 ± 5.48	37 ± 9.89	0.081*
	α vs c → *p* = 0.81**			
HβΑ1c (%)	8.15 ± 1.25	8.03 ± 1.71	9.06 ± 1.56	0.699*
	α vs b → *p* = 0.88**			
T1D duration (years)	7.56 ± 2.96	7.15 ± 3.27	6.35 ± 0.31.25	0.920*

b					
Sex	High protection	Ordinary protection	Low protection	X^2^	*p*
Boys	**31.2%**	**62.5%**	6.2%	0.929	0.628***
Girls	20%	60%	**20%**		
Paternal education				X^2^	*p*
Primary/middle	0%	**80%**	**20%**	4.191	0.381***
High-school/technical	**40%**	60%	0%		
University/postgraduate	33.3%	50%	16.6%		
Maternal education				X^2^	*p*
Primary/middle	25%	50%	**25%**	3.0	0.558***
High-school/technical	**40%**	60%	0%		
University /Postgraduate	16.6%	**66.7%**	16.6%		
Parental marital status				X^2^	*p*
Separated/divorced	0%	**66.7%**	34.4%	3.09	0.213***
Married	**33.3%**	61.1%	5.6%		

Bold = maximum proportions in each contraception subgroup

*Kruskal-Wallis one-way ANOVA

**Student’s T-test

***Pearson’s chi-square test

**Table 4:** Controls-level of contraception by demographic parameters

	HIGH protection (a) *n* = 15 (≥2 methods)	ORDINARY protection (b) *n* = 25 (1 method)	LOW Protection (c) *n* = 7 (no contraception or withdrawal)		*p*
Patient’s age (years)	16.47 ± 1.5	16.56 ± 1.29	16.14 ± 1.069		0.768*
Age at 1st sexual contact (years)	14.91 ± 2.16	15.53 ± 1.07	14.75 ± 1.33		0.417*
Father’s age (years)	47.60 ± 5.50	49.79 ± 7.41	45.0 ± 4.764		0.211*
Mother’s age (years)	42.80 ± 4.03	44.72 ± 5.05	44.0 ± 5.68		0.486*
Paternal education				X^2^	***p***
Primary/middle	**33%**	33.3%	**33.3%**	1.926	0.749***
Highschool/technical	31.8%	54.5%	13.6%		
University/postgraduate	26.6%	**60%**	13.3%		
Maternal education				**X**^**2**^	***P***
Primary/middle	28.6%	42.8%	**28.6%**	3.23	0.519***
Highschool/technical	**38.4%**	44.4%	16.6%		
University/postgraduate	21%	**68.4%**	10.5%		
Parental Marital Status				**X**^**2**^	***P***
Separated/divorced	10%	**98%**	0%	**6.334**	0.042***
Married	**34.3%**	45.7%	**20%**		
Gender				**X**^**2**^	***p***
Boys	**34.4%**	50%	**15.6%**	0.418	0.811***
Girls	26.6%	**60%**	13.3%		

Bold = maximum proportions in each contraception subgroup

*Krucall-Wallis one-way ANOVA

**Pearson’s chi-square test

Among T1D adolescents (Table 3a), the high protection group was characterized by younger patients’ age (16.5 yrs vs 17.0 yrs in the low protection group, *p* = 0.023), and by older age at 1st sexual contact (16.4 yrs vs 15.7 yrs in the ordinary protection group, *p* = 0.010) (Table 3a). The low protection group was characterized by older patients’ age (*p* = 0.023), and younger fathers’ age (41.0 yrs vs 51.92 yrs, *p* = 0.046) compared to the ordinary protection group. Also, although the mean maternal age was younger in the low protection group than the ordinary protection group (37.0 yrs vs. 47.6 yrs), the difference did not reach statistical significance (*p* = 0.081). No significant difference in the degree of contraception use according to parental education and marital status was observed in the patients’ group (Table 3b).

In the control group (Table 4), no significant difference was observed in the adolescents and their parents’ age, age at sexual debut or gender. However, regarding parental marital status, a significant difference among the 3 groups according to the degree of contraception use was observed, with the majority of adolescents using double contraception belonging to the married parents’ group (34.3 vs 10% in the divorced group) (x2 = 6.334, *p* = 0.042).

As the condom was the most frequent contraceptive method used in both study groups, logistic regression analysis was conducted with the use of condoms as the dependent variable in male controls, female controls, T1D males, and T1D females separately. Independent variables were age, disease duration, HbA1c, age at first intercourse, and parental educational level. No significant associations were found (*p* > 0.05). Greater age at first intercourse among male controls tended to be associated with a greater probability of using a condom during the most recent intercourse (OR = 3.67; 95% CI: 0.87–15.44, *p* = 0.077).

**Fig. 1:**
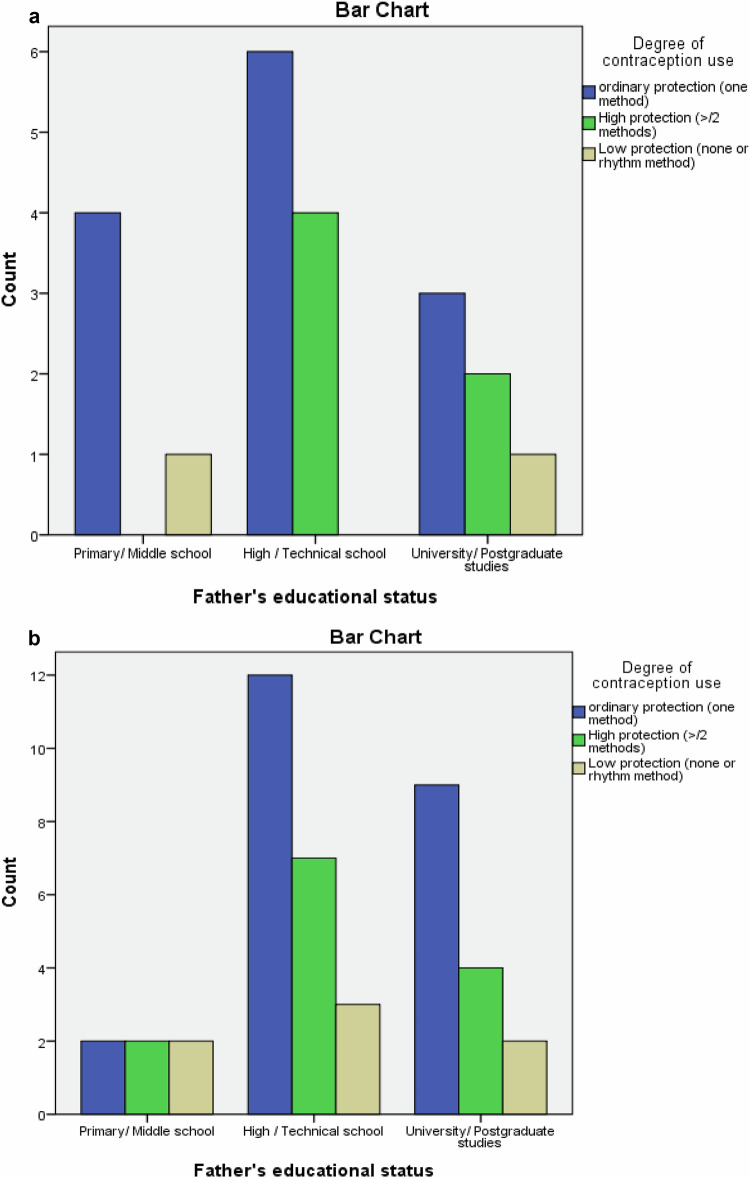
a. T1D adolescents-degree of contraception by paternal educational status. b. controls- degree of contraception by paternal educational status

In Fig. 1a and b, the prevalence of double, ordinary, and low contraception according to the level of paternal education (a. primary/secondary education, b. high-school/technical, c. university/postgraduate studies) are shown. In the lower paternal education group, the use of ordinary contraception was more frequent among T1D adolescents than controls. Moreover, in the group with high-school/technical parental education, the use of double contraception was more frequent among T1D patients than controls, and there were no patients with low protection in this subgroup.

## Discussion

The present study examines sexual knowledge and contraceptive use of T1D adolescents in comparison with their healthy peers. According to our understanding, minimal previous data are available on the use of contraceptives by T1D adolescents and on their knowledge of STDs and potential factors affecting their use. Furthermore, this is the first study of this kind in Greece.

T1D teens were not adequately informed about HIV as an STD at a significantly lower percentage than controls (76.4 vs. 89.6%, *p* = 0.027). A limited level of knowledge regarding other STDs was equally demonstrated in both groups. Possible explanations for the above could be that adolescents with T1D may not feel confident enough to discuss STDs or birth control with their health provider due to the fear of discrimination. Furthermore, clinicians do not usually discuss birth control unless the patient requires it, because discussion about problems of diabetic control is time-consuming. Impaired metabolic control is also associated with risky sexual behaviors and poor knowledge of STD prevention [12].

In the present study, the proportion of T1D adolescents using no protection at all was double that of controls (23.5 vs 10.2%, respectively). In other studies, the frequency of unprotected sex among T1D adolescents was even higher (29–39%) [3, 6, 13] and lower (1.5–8.2%) among non-diabetic teenagers [10, 11]. Girls with T1D may engage in unprotected sex because they mistakenly believe that they will have difficulty conceiving due to their diabetes [14, 15]. Additionally, peer pressure, lack of appropriate education and awareness, high levels of stress due to the underlying condition, or cultural and religious beliefs that discourage open discussion about sex and contraception can also prevent adolescents with T1D from using protection during sex [16, 17].

In the present study, the most common method of contraception was the male condom for both groups (T1D:85%, controls: 72.5%), whereas the second most preferred method for patients and controls was withdrawal, followed by the “rhythm” method, both offering low protection. However, fewer T1D adolescents (47.8%) than controls (51.9%) used condoms during every sexual intercourse. Even higher percentages of condom use were reported in previous studies, i.e., 73–97% of T1D adolescents and 84–95% of healthy counterparts [2, 3, 6]. These data suggest a preference for condom use, probably due to ease of implementation and low cost.

In previous studies, the use of contraceptive pills ranged from 23.7–52% of T1D adolescents [2, 3, 6] and 22.2–60% of controls [2, 6]. According to our findings, the use of birth control pills was significantly lower among both T1D and control adolescents (11.8 and 8.3%, respectively). The minimal use of oral contraceptives (OCs) among T1D adolescents could be associated with adverse metabolic effects, leading to the development of metabolic syndrome [18] or thromboembolic episodes [19]. However, according to a study by Cod et al., OCs with low ethinylestradiol concentration ( <35 μg) do not influence glycemic control [20, 21]. According to ISPAD and WHO recommendations, the use of OC in contraception is a safe choice for T1D adolescents [7, 22].

Although LARC has been reported as the contraceptive method with the lowest risk for thromboembolism in women with T1D [23], none of our patients and controls used LARC. Similarly, in another study, less than 5% of women aged 15–44 years used one of these methods [24]. The low rate of use of LARCs in adolescents is related to barriers to their use, including difficulties gaining access to insertion and the cost [25]. In addition, a lack of knowledge of the benefits of LARCs can result in misconceptions and low use frequency [26].

In our study, 35% of T1D adolescents and 27.7% of controls used a combination of condoms with birth control pills, withdrawal, or “rhythm method”. In contrast, in other studies, the use of combined contraceptive methods was higher, ranging from 7–30% in T1D adolescents and 44% in controls [2, 3]. Differences in double contraception use could be attributed to better education practices on sexual issues in different study populations.

In this study, adolescents with T1D and younger age reported higher use of contraception. This could be explained by the fact that younger adolescents are still closer to their parents and will seek advice from them, thus being more cautious in managing risky sexual behaviors. In contrast, older adolescents are more likely to be affected by their peers, the internet, and other sources of information, which in turn increases the risk of not using condoms [27]. The significant role of the family in sexual education in Greece, especially in younger adolescents, is still evident [28].

In comparison, the low contraception group in the present study was characterized by younger paternal and maternal ages. This has been linked to social norms, economic hardship, and lack of parental guidance on sexual health in families with young parents. On the other hand, older parental age is associated with more positive parent-child interaction, better parental psychological functioning, and better communication in terms of open contraception discussion [29, 30].

In the control group, adolescents with married parents reported more frequent contraception use than those with separated/divorced parents (*p* = 0.042). Additionally, the use of one contraceptive method was higher among patients than controls in the lower parental education group (Fig. 1a, b). As there are no relevant previous studies on T1D adolescents, the above findings could be compared with those in the general population; among healthy young women, double contraception has been observed more often in adolescents attending a private school, and those residing in urban areas [31, 32], or with parents having a college degree [33]. These results indicate that T1D adolescents were more responsible regarding contraception than controls and that parental counseling from parents with standard family structure and higher education was essential for both groups.

Emergency contraception (mini-pill or Depo-Provera) was used by 18.8% of T1D adolescents and 4.3% of controls in the present study. Similarly, higher percentages of T1D adolescents in other studies used progestin-only methods, with reported use in 23–38% of T1D adolescents and 10% of the controls [3, 6]. T1D adolescents may be more irresponsible in terms of unexpected pregnancies due to some reasons, including misconceptions of reduced fertility, the inclination for risky behaviors, the need for peer acceptance, and lack of or inadequacy of diabetes health care professional contraception routine counseling.

Following logistic regression analysis on the factors associated with the use of condoms as the most preferred contraceptive method, with age, disease duration, HbA1c, age at first intercourse and parental educational level as independent variables, it was found in male controls that older age at first intercourse tended to be associated with greater probability of using a condom during the most recent intercourse. Moreover, older age at sexual debut characterized patients who used double contraception. The above suggest that older adolescents are better informed and more responsible in sexual activity. Reported early sexual debut among teenagers has been previously associated with a lower likelihood of contraception use, heightening the risk of unexpected pregnancy and sexually transmitted diseases [34–37].

The present study has certain limitations, such as the small number of adolescents, and especially girls, in both groups with complete sexual relations, which did not allow the achievement of statistical significance on certain occasions. However, the study includes a representative population of Greek T1D adolescents, as it comes from the Diabetes Unit of a Tertiary University Children’s Hospital in Athens, which is the referral center for central and southern Greece and the islands. Moreover, another advantage of this study is that it includes a control population of healthy adolescents matched 1:2 for age, gender, educational and socioeconomic status, while most relative studies do not include control populations.

## Conclusions

This study concludes that both T1D adolescents and healthy peers limited levels of knowledge concerning sexual issues and the use of contraceptive methods. Both groups preferred the condom, followed by behavioral methods (withdrawal and rhythm). Older age at sexual debut was associated with a higher frequency of contraception use among healthy males and double contraception among T1D adolescents. Parental counseling from parents with a standard family structure and higher education was promoting the use of contraception. The above underline the necessity for health care professionals, in cooperation with parents, to provide sexual education and contraception counseling to young adolescents with T1D and their healthy peers.

## Supplementary information

Supplementary material

## Data Availability

The datasets used and analyzed during the current study are available from the corresponding author on reasonable request.
