# Supplementary material for: Sexual knowledge and contraceptive use in adolescents with type 1 diabetes in comparison with their healthy peers
Source: Endocrine. 2025 Aug 31;90(2):488–97. doi: 10.1007/s12020-025-04345-1 (PMC12572054; doi:10.1007/s12020-025-04345-1)
Supplement: Supplementary file 1 — Supplementary material [file 12020_2025_4345_MOESM1_ESM.docx]

**Appendices:**

**Table 1. Participants' knowledge of sexually transmitted diseases**

| **Which of the following is a Sexually Transmitted Disease?** | | Groups | | | | P |
| --- | --- | --- | --- | --- | --- | --- |
|  |  | Controls | | T1D | |  |
|  |  | N (%) | N (%) | N (%) | N (%) |  |
| Trichomonas | No | 95 | 82.6 | 45 | 81.8 | 0.957+ |
|  | Yes | 13 | 11.3 | 6 | 10.9 |  |
|  | Don’t know/ No answer | 7 | 6.1 | 4 | 7.3 |  |
| Chlamydia | No | 79 | 68.7 | 35 | 636 | 0.805+ |
|  | Yes | 29 | 25.2 | 16 | 29.1 |  |
|  | Don’t know/ No answer | 7 | 6.1 | 4 | 7.3 |  |
| Gonorrhea | No | 101 | 87.8 | 47 | 85.5 | 0.814++ |
|  | Yes | 7 | 6.1 | 4 | 7.3 |  |
|  | Don’t know/ No answer | 7 | 6.1 | 4 | 7.3 |  |
| Genital herpes | No | 55 | 47.8 | 23 | 41.8 | 0.758+ |
|  | Yes | 53 | 46.1 | 28 | 50.9 |  |
|  | Don’t know/ No answer | 7 | 6.1 | 4 | 7.3 |  |
| Syphilis | No | 53 | 46.1 | 20 | 36.4 | 0.488+ |
|  | Yes | 55 | 47.8 | 31 | 56.4 |  |
|  | Don’t know/ No answer | 7 | 6.1 | 4 | 7.3 |  |
| HIV/ AIDS | No | 5 | 4.3 | 9 | 16.4 | **0.027++** |
|  | Yes | 103 | 89.6 | 42 | 76.4 |  |
|  | Don’t know/ No answer | 7 | 6.1 | 4 | 7.3 |  |
| Hepatitis A | No | 85 | 73.9 | 35 | 63.6 | 0.372+ |
|  | Yes | 23 | 20.0 | 16 | 29.1 |  |
|  | Don’t know/ No answer | 7 | 6.1 | 4 | 7.3 |  |
| Hepatitis B | No | 81 | 70.4 | 31 | 56.4 | 0.180+ |
|  | Yes | 27 | 23.5 | 20 | 36.4 |  |
|  | Don’t know/ No answer | 7 | 6.,1 | 4 | 7.3 |  |
| Hepatitis C | No | 88 | 76.5 | 37 | 67.3 | 0.422+ |
|  | Yes | 20 | 17.4 | 14 | 25.5 |  |
|  | Don’t know/ No answer | 7 | 6.1 | 4 | 7.3 |  |
| Human papillomavirus (HPV) |  | 90 | 78.3 | 42 | 76.4 | 0.947+ |
|  | Yes | 18 | 15.7 | 9 | 16.4 |  |
|  | Don’t know/ No answer | 7 | 6.1 | 4 | 7.3 |  |

+Pearson’s x^2^ test ++Fisher’s exact test

**Figure 2a. T1D ADOLESCENTS-DEGREE OF CONTRACEPTION BY GENDER**


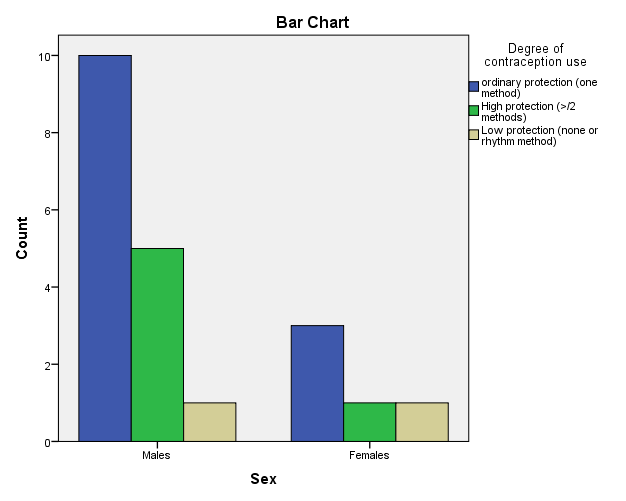


**Figure 2b. CONTROLS-DEGREE OF CONTRACEPTION BY GENDER**

**
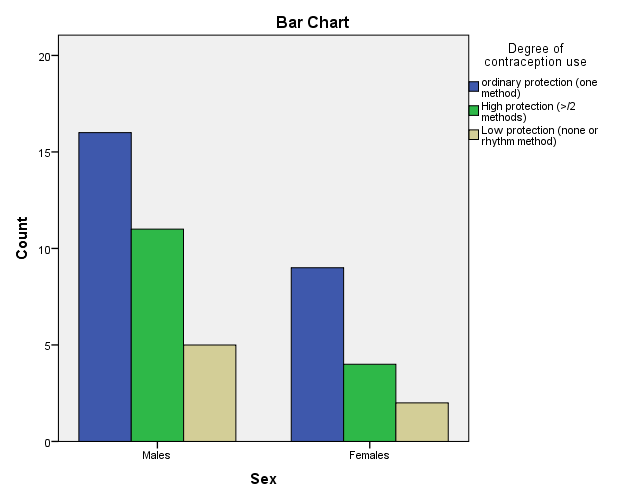
**

In Figures 2a and 2b, the prevalence (histograms) of double contraception, ordinary (1 method), and low contraception by gender among T1D adolescents and controls are shown. All degrees of protection were used at a much lower frequency among T1D female adolescents than among males, and the same was observed in the control group. Moreover, double and ordinary protection were more frequent among males than females for both study groups

**Figure 3a.** **T1D ADOLESCENTS**-**DEGREE OF CONTRACEPTION BY PARENTAL FAMILY STATUS.**

**
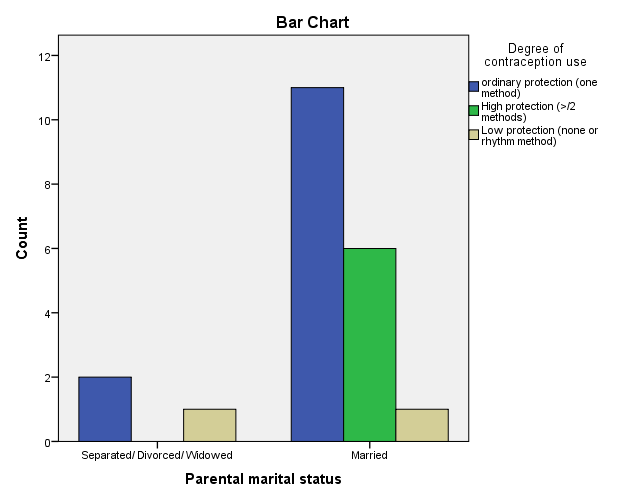
**

**Figure 3b.** **CONTROLS**-**DEGREE OF CONTRACEPTION BY PARENTAL FAMILY STATUS.**


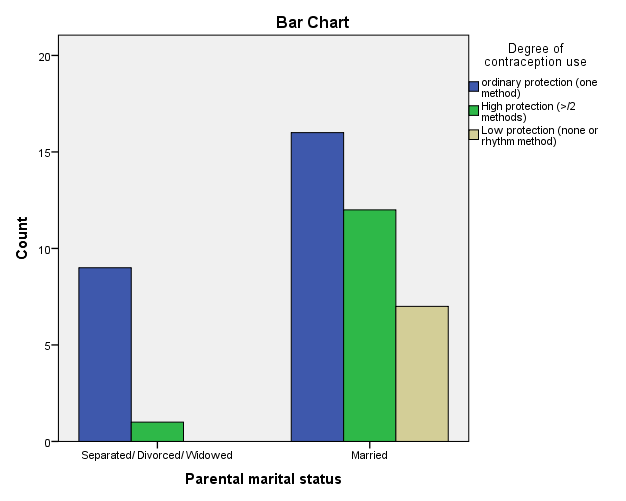


In Figures 3a and 3b, among T1D adolescents whose parents were divorced/widowed the frequencies of different degrees of contraception were lower than the relative ones of the control group.
